# Crystal Structure of Allantoinase from *Escherichia coli* BL21: A Molecular Insight into a Role of the Active Site Loops in Catalysis

**DOI:** 10.3390/molecules28020827

**Published:** 2023-01-13

**Authors:** Yen-Hua Huang, Po-Chun Yang, En-Shyh Lin, Ya-Yeh Ho, Wei-Feng Peng, Hsin-Pin Lu, Chien-Chih Huang, Cheng-Yang Huang

**Affiliations:** 1 Department of Biomedical Sciences, Chung Shan Medical University, Taichung City 402, Taiwan; 2 Department of Beauty Science, National Taichung University of Science and Technology, Taichung City 403, Taiwan; 3 Department of Medicine, College of Medicine, Chung Shan Medical University, Taichung City 402, Taiwan; 4 Department of Pediatrics, National Taiwan University Children’s Hospital, Taipei 100, Taiwan; 5 Department of Medical Research, Chung Shan Medical University Hospital, Taichung City 402, Taiwan

**Keywords:** allantoinase, hydantoinase, dihydropyrimidinase, active site loop, industrial enzyme

## Abstract

Allantoinase (ALLase; EC 3.5.2.5) possesses a binuclear metal center in which two metal ions are bridged by a posttranslationally carbamylated lysine. ALLase acts as a key enzyme for the biogenesis and degradation of ureides by catalyzing the conversion of allantoin into allantoate. Biochemically, ALLase belongs to the cyclic amidohydrolase family, which also includes dihydropyrimidinase, dihydroorotase, hydantoinase (HYDase), and imidase. Previously, the crystal structure of ALLase from *Escherichia coli* K-12 (EcALLase-K12) was reported; however, the two active site loops crucial for substrate binding were not determined. This situation would limit further docking and protein engineering experiments. Here, we solved the crystal structure of *E. coli* BL21 ALLase (EcALLase-BL21) at a resolution of 2.07 Å (PDB ID 8HFD) to obtain more information for structural analyses. The structure has a classic TIM barrel fold. As compared with the previous work, the two missed active site loops in EcALLase-K12 were clearly determined in our structure of EcALLase-BL21. EcALLase-BL21 shared active site similarity with HYDase, an important biocatalyst for industrial production of semisynthetic penicillin and cephalosporins. Based on this structural comparison, we discussed the functional role of the two active site loops in EcALLase-BL21 to better understand the substrate/inhibitor binding mechanism for further biotechnological and pharmaceutical applications.

## 1. Introduction

**Figure 1:**
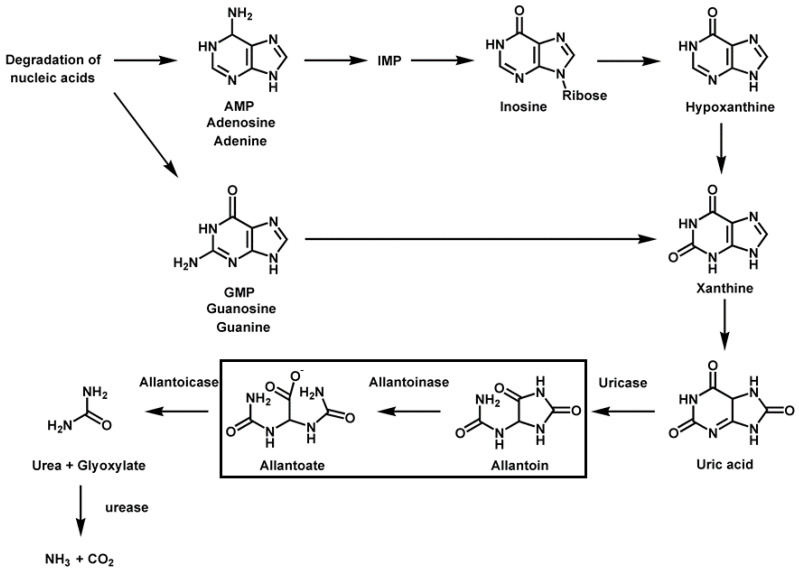
The pathways of purine nucleotide degradation. The chemical reaction catalyzed by allantoinase is highlighted. Allantoinase is a key enzyme for allantoin degradation. In the present study, we solved the crystal structure of *E. coli* BL21 allantoinase (EcALLase-BL21) at a resolution of 2.07 Å (PDB ID 8HFD) to obtain more information for structural analyses.

Allantoinase (ALLase; EC 3.5.2.5) plays an essential role in the catabolism pathway for purine degradation [1]. ALLase occurs in a wide variety of organisms, including bacteria, fungi, and plants, and a few animals. ALLase catalyzes the reversible conversion of allantoin to allantoic acid by hydrolytic cleavage of the five-member hydantoin ring (Figure 1). This ALLase-catalyzed reaction is a key process in the biosynthesis and degradation of ureide required for the utilization of nitrogen in purine-derived compounds [2]. ALLase is a homotetrameric dinuclear metalloenzyme [3,4], but some ALLases initially annotated as polysaccharide deacetylases are metal independent [5]. Thus, even without the ALLase gene, some bacteria can still use allantoin to utilize nitrogen.

Allantoin in plants participates in many positively signaling regulations and is also a signaling chemical that conveys information on local conditions in plant–plant interactions [6]. For example, allantoin enhances drought tolerance in rice [7]. In addition, allantoin also positively affects salt tolerance by increasing the reactive oxygen species scavenging capacity and maintaining sodium and potassium homeostasis [8]. ALLase is required for flux of fixed nitrogen to ureides in bean nodules [9]. Allantoin is abundant in many medical plants and has the pharmaceutical potential to be used in IgE-independent anti-allergic and anti-inflammatory therapies [10,11,12]. Allantoin is one of the most important botanicals pertaining to dermatologic uses [13]; therefore, understanding the enzymatic reaction of ALLase is of considerable interest for further biotechnological applications.

ALLase [14] is a member of the cyclic amidohydrolase family [15,16], which also includes dihydroorotase (DHOase) [17,18,19,20,21,22,23], dihydropyrimidinase (DHPase) [24,25,26,27,28,29,30,31], hydantoinase (HYDase) [32,33,34], and imidase [35,36,37]. Almost all these enzymes contain the binuclear metal center that consists of four His, one Asp, and one posttranslational carbamylated Lys (Kcx) residues [15]. Some of these amidohydrolases have been suggested as chemotherapeutic targets for anticancer, antimicrobial, and antimalarial drug development because of their involvement in the key reactions of nucleotide biosynthesis [15]. Thus, it is worth exploiting the new inhibitors against these targets for drug development.

Recently, we found that the extract of *Nepenthes miranda* was capable of inhibiting ALLase [38], DHOase [39], and DHPase [40] activities. Through gas chromatography–mass spectrometry analysis (GC–MS), the active ingredients abundant in this extract were detected and identified [38]. Given that the crystal structure of ALLase from *Escherichia coli* K-12 (EcALLase-K12) [41] is available, the top six compounds in this *N. miranda* extract, namely, plumbagin, lupenone, palmitic acid, stigmast-5-en-3-ol, neophytadiene, and citraconic anhydride, were docked to EcALLase-K12 [38]. These compounds might be useful alone or in combination for further inhibitor optimization and structure-based drug design for anti-ALLase activity.

**Figure 2:**
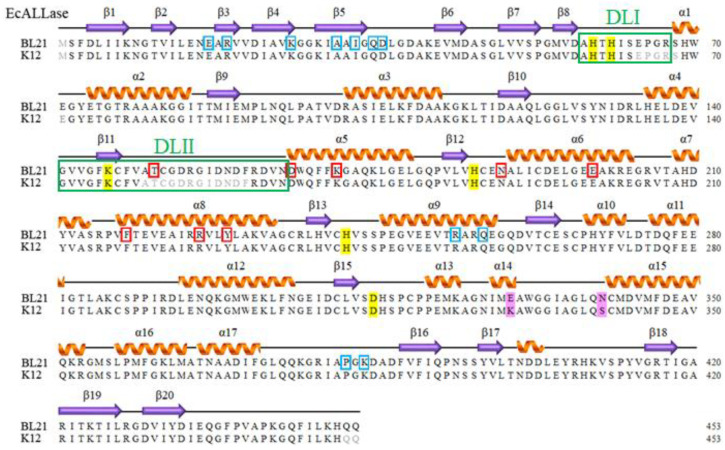
Sequence alignment of EcALLase-BL21 and EcALLase-K12. The secondary structural elements of EcALLase-BL21 are shown with the sequence. The active site dynamic loop I (DLI; A58–R67) and dynamic loop II (DLII; G141–N165) are boxed in green. The unobserved residues 64–68, 150–161, and 452–453 in EcALLase-K12 ternary structure were colored in grey. In comparison, these residues in the structure of EcALLase-BL21 were clearly found. The metal-binding sites are shaded in yellow. The amino acids that are involved in monomer–monomer interface via hydrogen bonding are boxed in red. The amino acids that are involved in dimer–dimer interface via hydrogen bonding are boxed in blue. In comparison, only two residues of EcALLase-K12, i.e., K330 and S340, are different from and replaced with E330 and N34 in EcALLase-BL21 (shaded in pink).

It is well established that the two dynamic loops in the active site of DHPase [15], HYDase [33], and DHOase [42] are crucial for substrate entrance and product release. HYDase is an important enzyme used for hydrolysis of chemically synthesized cyclic hydantoins for industrial production of antibiotics such as semisynthetic penicillin and cephalosporins [43,44]. The two active site loops important for substrate recognition, the segments of amino acid residues (aa) D62–D72 and M153–D162 in *Bacillus stearothermophilus* HYDase (BsHYDase), correspond to segments aa A58–R67 and G141–N165 in EcALLase-K12 (boxed in green in Figure 2). However, these two dynamic loops were not observed in the ternary structure of EcALLase-K12. To better study the substrate binding, substrate specificity, and catalytic mechanism of ALLase, knowing the structure of these two important loops is highly desirable for further drug design and pharmaceutical applications of ALLase. In addition, these missed loops in the structure of EcALLase-K12 will significantly affect the docking results and protein engineering strategy.

*E. coli* K-12 and BL21 have been the subjects of classical experiments from which much of our understanding of molecular genetics has emerged [45]. In this study, we solved the crystal structure of *E. coli* BL21 ALLase (EcALLase-BL21) at a resolution of 2.07 Å (PDB ID 8HFD) to obtain more information for structural analyses. The two missed active site loops in EcALLase-K12 were clearly determined in our structure of EcALLase-BL21. This EcALLase-BL21 structure gives full structural information and therefore might be more suitable for further docking and protein engineering experiments.

## 2. Results

### 2.1. Sequence Analysis of EcALLase-BL21

*E. coli* BL21(DE3) is widely used to express recombinant proteins. This study aimed to investigate the structure of EcALLase-BL21 for further pharmaceutical applications such as drug design and biotechnology development. The gene *ECD_00462*, encoding EcALLase-BL21, was initially found in a database of the National Center for Biotechnology Information (NCBI) [46]. Based on the known nucleotide sequence, the predicted EcALLase-BL21 monomer has a length of 453 amino acid residues and a molecular mass of 49.6 kDa. EcALLase-BL21 has a calculated pI value of 5.12 via the amino acid sequence. Six essential residues, H59, H61, Kcx146, H186, H242, and D315, participate in the assembly of a binuclear metal center within the active site of EcALLase-K12 [41], and are also found in EcALLase-BL21 (Figure 2). In comparison, only two residues of EcALLase-K12, i.e., K330 and S340, are different from and replaced with E330 and N34 in EcALLase-BL21. Although the crystal structure of EcALLase-K12 was solved, many regions, especially several active site residues, were not determined. Due to its sequence similarity, EcALLase-BL21 was selected to obtain the whole structural information of ALLase.

### 2.2. Crystallization and Data Collection of EcALLase-BL21

Prior to this study, the crystal structure of the EcALLase from subspecies K-12 was available for computational use (PDB ID 3E74) [41]. However, three segments, i.e., aa 64–68, 150–161, and 452–453, in the ternary structure of EcALLase-K12 were undetermined [41]. The region aa 150–161 may contain several important residues for substrate binding (Figure 2). Therefore, this situation certainly limited binding model prediction of the position the inhibitor of ALLase occupies as the molecular docking is performed. Given that this active site loop is unobserved in the structure of EcALLase-K12, we attempted to determine the structure of another EcALLase, namely EcALLase-BL21, for inhibitor docking experiments. Crystals of EcALLase-BL21 were grown at room temperature by hanging drop vapor diffusion in 20% PEG8000 and 100 mM CHES at pH 9.5. These crystals reached full size in 7–13 days. Data were collected using an EIGER2 X 16M Detector at an SPXF beamline TPS 07A of the National Synchrotron Radiation Research Center (NSRRC). The data completeness was over 99% (Table 1).

### 2.3. Overall Structure of EcALLase-BL21

**Figure 3:**
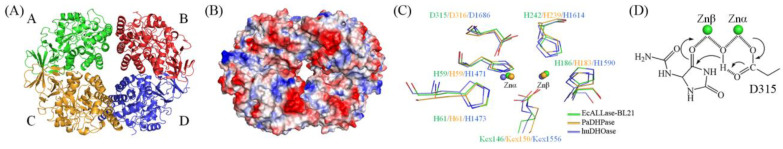
Structure of EcALLase-BL21. (**A**) Ribbon diagram of EcALLase-BL21 tetramer. Each ScDHOase monomer is color coded. Two zinc ions in the active site are presented as black spheres. (**B**) The electrostatic potential surface of EcALLase-BL21. Tetramerization of EcALLase-BL21 monomers generated a central cavity but it is unclear what function, if any, it was created for. (**C**) Superposition of the active site of members of the cyclic amidohydrolase family. Their active sites contain four His, one Asp, and one Kcx, which are required for di-metal binding and catalytic activity. EcALLase-BL21 (PDB ID 8HFD; green), PaDHPase (PDB ID 5E5C; light orange), and huDHOase (PDB ID 8GW0; slate) are shown. The architecture of these active sites is similar. (**D**) The proposed mechanism of EcALLase-BL21 for catalysis. The hydrolysis of allantoin likely undergoes three steps: the hydrolytic water molecule must be activated for nucleophilic attack, the amide bond of the substrate must be made more electrophilic by polarization of the carbonyl–oxygen bond, and then the leaving-group nitrogen must be protonated as the carbon–nitrogen bond is cleaved. The metal ions are shown as green circles.

We crystallized EcALLase-BL21 and determined its structure at a resolution of 2.07 Å (Table 1). The crystal of EcALLase-BL21 belonged to space group C_1_2_1_, with cell dimensions of *a* = 203.16, *b* = 77.14, and *c* = 144.85 Å. Four monomers of EcALLase-BL21 per asymmetric unit were present (Figure 3A). Most of the electron density for EcALLase-BL21 was of good quality and no discontinuity was observed. The global architecture of EcALLase-BL21 revealed a TIM-barrel structure that consisted of 17 α-helices, 20 β-sheets, and 2 Zn ions. The electrostatic potential surface of EcALLase-BL21 (Figure 3B) shows that the subunit–subunit interactions are stabilized by the charge–charge interactions at each subunit interface. Tetramerization of EcALLase-BL21 monomers generated a central cavity, but it is unclear what function, if any, it was created for (Figure 3B). The active site of EcALLase-BL21 containing H59, H61, Kcx146, H186, H242, and D315, which were required for the metal binding, was similar to that of EcALLase-K12 and other members of the cyclic amidohydrolase family (Figure 3C), such as *Pseudomonas aeruginosa* DHPase (PaDHPase) and the dihydroorotase domain of human CAD protein (huDHOase). Accordingly, these cyclic amidohydrolases might use a similar mechanism for catalysis (Figure 3D) [15].

### 2.4. Dynamic Loops of EcALLase-BL21

**Figure 4:**
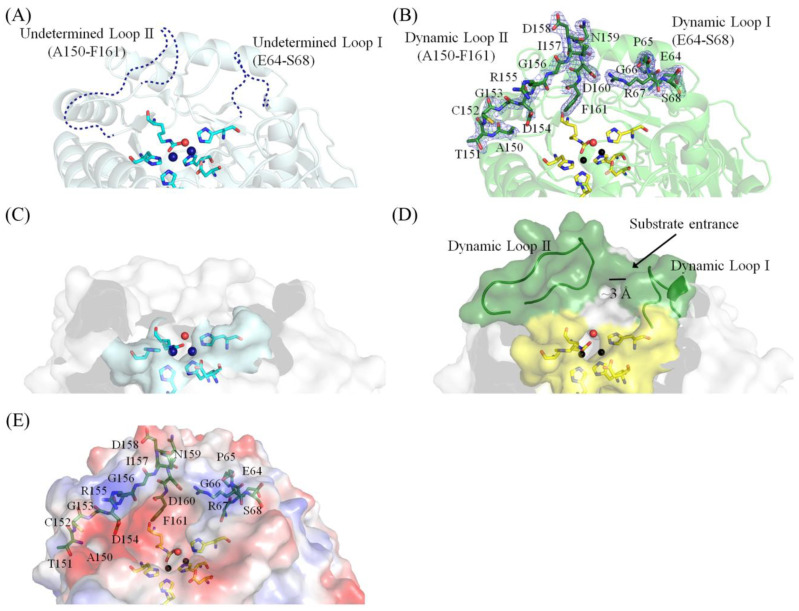
Dynamic loops and the tunnel bottleneck of EcALLase-BL21. (**A**) The active site of EcALLase-K12. The two active site loops crucial for substrate binding were not determined. These two dynamic loops in EcALLase-K12 are segments A58–R67 (loop I) and G141–N165 (loop II). When we used the structure of EcALLase-K12 for inhibitor docking and sketching the chemical mechanism, we found that it was difficult to draw a correct conclusion, probably due to the lack of structure for these two active site loops. (**B**) The active site of EcALLase-BL21. The electron density of the residues on the two dynamic loops in our structure of EcALLase-BL21 was well defined. (**C**) The tunnel bottleneck of EcALLase-K12. Because the structure of the two loops was not solved, the cross-section bottleneck size for substrate entrance could not be determined. (**D**) The tunnel bottleneck of EcALLase-BL21. The bottleneck size of the active site pocket of EcALLase-BL21 was approximately 3 Å. Given that the tunnel bottleneck (~3 Å) is narrower than the size of substrate allantoin (6–9 Å), this structure may be the closed form of EcALLase-BL21. (**E**) The electrostatic potential surface of the structure of EcALLase-BL21. Residues on the dynamic loops are indicated.

Based on their similar binuclear metal active site architecture and post-carbamylation, ALLase, DHOase, DHPase, and HYDase share several enzymatic properties and may use a similar mechanism for catalysis [47,48]. HYDase is an industrial enzyme [44,49], which has been highlighted for its capacity for biotechnological and pharmaceutical applications in the production of unnatural amino acids. It is well established that DHOase [39,42,50], DHPase [30,31,51], and HYDase [33,43,52] have flexible dynamic loops directly involved in substrate binding, product release, and stabilization of the transition state. This type of dynamic loop has not yet been mentioned and identified in ALLase. Correspondingly, the two dynamic loops in EcALLase-K12 should be A58–R67 (loop I) and G141–N165 (loop II). When we used the structure of EcALLase-K12 for inhibitor docking and sketching the chemical mechanism, we found that it was difficult to draw a correct conclusion, probably due to the lack of structure for these two active site loops (Figure 4A). Therefore, we solved the crystal structure of EcALLase-BL21 to better understand the structure–function relationship of ALLase. This structural information of EcALLase may also be useful for production of other unnatural amino acids when manipulating and engineering these two active site loops to modulate and change the substrate specificity for obtaining a new product [52]. As compared to the crystal structure of EcALLase-K12, these active loops in our structure, as solved in this study, were fully determined (Figure 4B). Accordingly, the position information of these active site residues can be used for further docking and protein engineering experiments.

These two dynamic loops that remain unsolved in the structure of EcALLase-K12 (Figure 4C) but appear in the current EcALLase-BL21 structure (Figure 4D,E) may be used for determining the catalytic state of this enzyme. It is known that the cross-section bottleneck size of the tunnel in DHPases can determine the open (for substrate binding) or closed (product release) form of the enzyme. For example, the tunnel bottlenecks created by these two dynamic loops in the open and closed forms of the DHPase are approximately 10 and 3 Å, respectively [31]. Based on our structure, the bottleneck size of the active site pocket of EcALLase-BL21 was approximately 3 Å (Figure 4D). Given that the tunnel bottleneck (~3 Å) is narrower than the size of substrate allantoin (6–9 Å), this structure may be the closed form of EcALLase-BL21. In addition, the basically charged residues on these loops may mediate the substrate entrance (Figure 4E). However, this speculation must be further elucidated biochemically and structurally.

### 2.5. Molecular Docking

**Figure 5:**
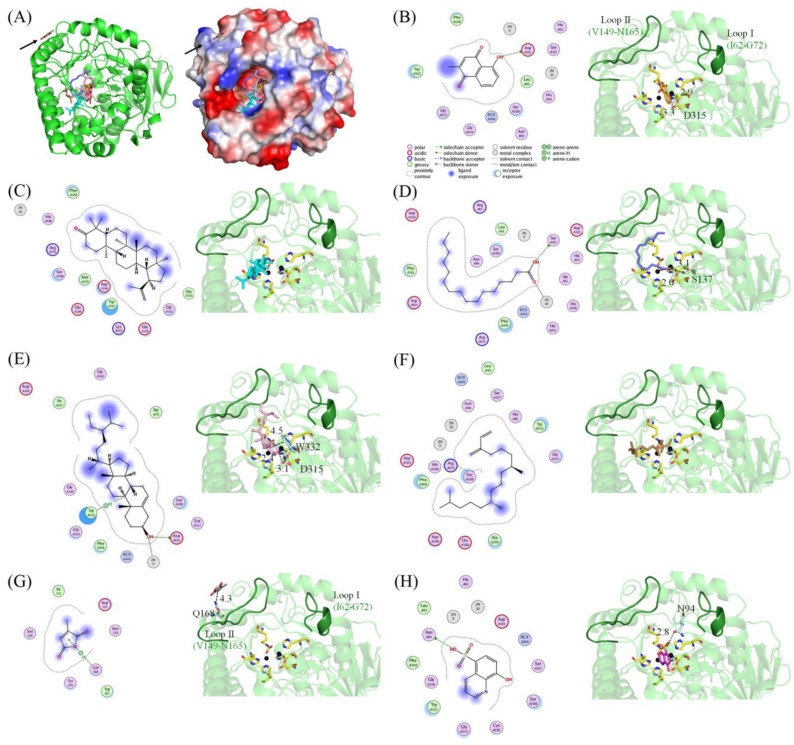
Molecular docking. (**A**) Docking results. Plumbagin, lupenone, palmitic acid, stigmast-5-en-3-ol, neophytadiene, citraconic anhydride, and 8-HQSA were docked to EcALLase-BL21 (PDB ID 8HFD) via the MOE-Dock tool. The superimposed structures of these EcALLase-BL21 complexes based on docking experiments are shown. As indicated, only citraconic anhydride could not be docked into the active site of EcALLase-BL21. The binding mode of (**B**) plumbagin, (**C**) lupenone, (**D**) palmitic acid, (**E**) stigmast-5-en-3-ol, (**F**) neophytadiene, (**G**) citraconic anhydride, and (**H**) 8-HQSA to EcALLase-BL21 is shown.

ALLase-catalyzed reaction is a key process in the biosynthesis of ureide [53], which is required for the utilization of nitrogen in purine-derived compounds, and, thus, ALLase may be a cytotoxic target. Recently, we have identified that the leaf extract of *N. miranda* possesses anti-ALLase activity [38]. Through GC–MS, the 16 most abundant ingredients in this extract were identified. The top six compounds [38] were plumbagin, lupenone, palmitic acid, stigmast-5-en-3-ol, neophytadiene, and citraconic anhydride. Given that a certain abundant compound in *N. miranda* may be responsible for inhibiting the activity of ALLase, their binding modes should be elucidated (Figure 5A). Along with the binding poses of plumbagin (Figure 5B), lupenone (Figure 5C), palmitic acid (Figure 5D), stigmast-5-en-3-ol (Figure 5E), neophytadiene (Figure 5F), and citraconic anhydride (Figure 5G) to EcALLase-BL21, the binding capacities of EcALLase-BL21 were also calculated via the MOE (molecular operating environment)-Dock tool [54]. Through MOE-Dock, receptor–ligand binding affinities with all possible binding geometries could be predicted on the basis of the docking score (the S score). These docking results using the structure of EcALLase-BL21, however, significantly differed from those obtained when the structure of EcALLase-K12 was used [38]. 8-Hydroxyquinoline-5-sulfonic acid (8-HQSA), an inhibitor of EcALLase [46], was also docked to EcALLase-BL21 (Figure 5H and Table 2). The docking result showed that, except for citraconic anhydride (Figure 5A), all other compounds could be docked into the active site pocket of EcALLase-BL21. In comparison with previous work, in that study only plumbagin could be docked into the active site of EcALLase-K12 [38]. These obvious differences may have resulted from the incomplete active site loops in the structure of EcALLase-K12 [41]. Based on the S scores, the binding capacity of these compounds was in the following order: stigmast-5-en-3-ol > lupenone > 8-HQSA > palmitic acid > plumbagin > neophytadiene > citraconic anhydride (Table 2). Accordingly, stigmast-5-en-3-ol, possessing the highest S score, might exhibit the greatest binding affinity to ALLase-BL21 among these selected compounds. To further elucidate how ALLase-BL21 can be suppressed by these possible inhibitors, the complexed crystal structure of ALLase-BL21 is highly desired.

### 2.6. Analysis of Substrate Binding Pockets of HYDase and ALLase

**Figure 6:**
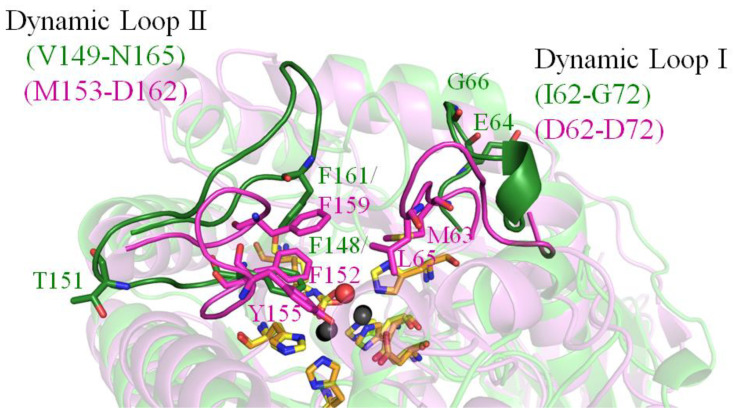
Analysis of substrate binding pockets of HYDase and ALLase. HYDase catalyzing the cleavage of the cyclic amide bond of 5′-monosubstituted hydantoins is widely employed for the commercial production of optically pure D- and L-amino acids for pharmaceutical applications. Like ALLase, HYDase also has two dynamic active site loops crucial for catalysis. The dynamic loop I and II (peach) are segments D62–D72 and M153–D162 in BsHYDase, respectively. Although HYDase and ALLase are similar enzymes and may use a similar mechanism for catalysis, their substrate specificities do not overlap. The active site loop residues M63, L65, F152, Y155, and F159 in BsHYDase constitute a hydrophobic lid to interact with the exocyclic substituent of the substrate hydroxyphenylhydantoin. The corresponding residues in EcALLase-BL21 are E64, G66, F148, T151, and F161. Given that the substrate specificity of HYDase can be rationally modulated by manipulating those residues on these two dynamic loops, changing the corresponding residues in EcALLase-BL21 may create a new ALLase for producing a series of unnatural amino acids for pharmaceutical applications.

HYDase catalyzing the cleavage of the cyclic amide bond of 5′-monosubstituted hydantoins is widely employed for the commercial production of optically pure D- and L-amino acids for pharmaceutical applications [44]. Although HYDase and ALLase are similar enzymes (Supplementary Figure S1) and may use a similar mechanism for catalysis, their substrate specificities do not overlap [14]. Like the EcALLase-BL21 examined in this study, HYDase also has two dynamic loops as crucial determinants of the substrate specificities [52]. Structural analysis indicated that the active site loop residues M63, L65, F152, Y155, and F159 in BsHYDase (PDB ID 1K1D) constitute a hydrophobic lid to interact with the exocyclic substituent of the substrate hydroxyphenylhydantoin. The corresponding residues in EcALLase-BL21 are E64, G66, F148, T151, and F161 (Figure 6). Given that the substrate specificity of HYDase can be rationally modulated by manipulating those residues on these two dynamic loops [52], changing the corresponding residues in EcALLase-BL21 may create a new ALLase for producing a series of unnatural amino acids for pharmaceutical applications. Further studies can directly focus on this issue for ALLase applications.

## 3. Discussion

The goal of the present work was to acquire a crystal structure of EcALLase for further drug design, docking and binding analyses, and substrate specificity studies. The EcALLase-K12 structure had been solved, but three segments, including the two active site loops, namely aa 64–68 and 150–161, in the ternary structure, were undetermined [41]. This situation therefore significantly affected the docking results and protein engineering strategy (Figure 5). This is why we tried to obtain a crystal of EcALLase-BL21 and determine its crystal structure (Figure 3). In this study, these two active site loops of EcALLase-BL21 were fully solved structurally (Figure 4). Thus, this current EcALLase structure may be more suitable for use in further docking and product design experiments (Figure 6).

Multidrug-resistant pathogenic bacteria are spreading rapidly worldwide and could become untreatable [55]. Given that the catabolism of allantoin (purine) is a nitrogen source in *E. coli* [56] and other microorganisms [57], and the utilization of allantoin as a nitrogen source is recognized as very important for bacterial virulence [58], blocking the activity of ALLase would be detrimental to bacterial survival. In addition, ALLase is not found in humans. Thus, ALLase may be a promising therapeutic target for developing antibiotics. Although 8-HQSA is obviously capable of inhibiting the activity of EcALLase-BL21 [46], this chelator may be harmful to human health. Recently, we found that extracts of *N. miranda* exhibit antibacterial and anti-ALLase activities [38]. More plant-derived products acting as active antibacterial and anti-ALLase agents for human health care should be worth further determination.

Optically pure D-p-hydroxylphenylglycine and its derivatives as side-chain precursors are required for industrial production of semisynthetic penicillin and cephalosporins [43,44]. To obtain these precursors, HYDase is used for hydrolysis of chemically synthesized cyclic hydantoins [44]. In this study, we determined the crystal structure of EcALLase-BL21 at a resolution of 2.07 Å (Table 1). Despite an undetectable similarity in the amino acid sequence, EcALLase-BL21 and HYDase share a structural resemblance. Although the catalytic chemistry is conserved [15], their substrate specificity does not overlap [14]. Since allantoin is a kind of hydantoin, we thought that ALLase might also be a useful enzyme for production of optically pure D-amino acids for medical applications. The structure of the two active site loops solved in this study may provide molecular insight into how allantoin, as well as other hydantoin with novel exocyclic substituents, can be recognized and hydrolyzed by EcALLase-BL21 and its engineered enzymes (Figure 4).

On the two dynamic loops of BsHYDase, the residues M63, L65, F152, Y155, and F159 interact with the exocyclic substituent of hydantoin [33]. Protein engineering of these residues had a significant effect on the substrate specificity of HYDase [52]. For example, the substrate specificity of the mutant F159A toward aromatic substrate hydroxyphenylhydantoin was enhanced by approximately 200-fold compared to that of the wild-type HYDase. In addition, saturation mutagenesis at this position (F159) revealed that the catalytic constant of the HYDase for aromatic substrates increased gradually as the size of the amino acid side chain decreased [52]. Correspondingly, these residues in EcALLase-BL21 are E64 (loop I), G66 (loop I), F148 (loop II), T151 (loop II), and F161 (loop II). Accordingly, these amino acid residues on the dynamic loops may be also critical and can be modulated by rational design for the substrate specificity of EcALLase-BL21. However, this speculation must be investigated and confirmed experimentally and structurally.

Previously, we solved complexed crystal structures of DHPase and DHOase with the anticancer drug 5-fluorouracil (5-FU) [20,24,26]. 5-FU is an FDA-approved drug with a remarkable therapeutic effect for the systemic treatment of cancers of the gastrointestinal tract, breast, head, and neck in the clinic [59]. 5-FU can bind to the active site of DHPase and DHOase. Based on the active sites’ similarity among ALLase, DHPase, and DHOase, we may therefore tentatively speculate about the binding capacity of ALLase to 5-FU. Currently, our laboratory is attempting to obtain crystals of the ALLase-5-FU complex for this investigation.

The crystal structures of HYDase [33], DHPase [24,27,28,29,31], DHOase [50,60,61], and ALLase (this study) revealed that the two active site dynamic loops are crucial for catalysis. The movement of the dynamic loop (corresponding to loop II of EcALLase-BL21) in DHOase was proposed as a part of the catalytic cycle [61]. Crystal structures of ligand-bound *E. coli* DHOase (EcDHOase) show that the flexible loop extends toward the active site when the substrate is bound (loop-in mode) or moves away from the active site, facilitating the product release (loop-out mode). EcDHOase also binds non-substrate ligands such as the product-like inhibitor 5-fluoroorotate through loop-out mode [61]. However, several recent structures of ScDHOase in complexes with different non-substrate ligands all indicated the loop-in mode, suggesting that this previous conclusion reached with EcDHOase may be species-dependent [18,19,20,39]. Regardless of their different binding modes, this flexible loop in DHPases and DHOases, perhaps including ALLase now, was crucial for the catalysis [15]. Thus, this dynamic loop in these amidohydrolases should be suitable drug targets for inhibiting the pyrimidine and purine metabolism selectively [40]. Accordingly, we propose that a compound that could partially occupy the active site and stably lock off the loop movement of EcALLase-BL21 would be a good candidate as an antibacterial drug lead for developing a potent inhibitor toward ALLase. The complexed structures of ALLase still need to be solved and obtained to better understand the substrate/inhibitor binding mechanism for further biotechnological uses.

## 4. Materials and Methods

### 4.1. Protein Expression and Purification

Based on the complete genome sequence of *E. coli* BL21 [45], the plasmid for EcALLase-BL21 (*allB*; *ECD_00462*) expression was constructed [46]. The expression vector pET21b-EcALLase-BL21 [46] was transformed into *E. coli* BL21 (DE3) cells and grown in LB medium at 37 °C. The overexpression was induced by incubating with 1 mM isopropyl thiogalactopyranoside at 25 °C for 9 h. Recombinant zinc-amended EcALLase-BL21 [3] was purified from the soluble supernatant by using Ni^2+^-affinity chromatography. The recombinant protein was eluted with a linear imidazole gradient and dialyzed against a dialysis buffer (20 mM Tris-HCl and 0.1 M NaCl, pH 7.9). Approximately 12 mg of purified EcALLase-BL21 was obtained from 1 L of an *E. coli* cell culture. The protein purity remained at >97% as determined using SDS–PAGE.

### 4.2. Crystallization Experiments

Before crystallization, the purified EcALLase-BL21 was concentrated to 30 mg/mL. Crystals of EcALLase-BL21 were grown at room temperature by hanging drop vapor diffusion in 20% PEG8000 and 100 mM CHES at pH 9.5. These crystals reached full size in 7–13 days. The crystals were transferred from a crystallization drop into the cryoprotectant solution (2 μL) with precipitant solution containing glycerol (25–30%) for a few seconds, mounted on a synthetic nylon loop (0.1–0.2 mm), flash cooled in liquid N_2_, and validated in an SPXF beamline TPS 07A of the NSRRC (Hinchu, Taiwan).

### 4.3. X-Ray Diffraction Data and Structure Determination

Data were collected using an EIGER2 X 16M Detector at an SPXF beamline TPS 07A at the NSRRC. Data sets were indexed, integrated, and scaled by HKL-2000 [62] and XDS [63]. The initial phase, density modification, and model building were performed using the AutoSol program [64] in the PHENIX. The iterative model building and structure refinement were performed using Refmac in the CCP4 software suite [65] and phenix.refine in the PHENIX software suite [66]. The initial phase of EcALLase-BL21 was determined through the molecular replacement software Phaser MR [67] by using EcALLase-K12 [41] as a search model. The correctness of the stereochemistry of the models was verified using MolProbity [68]. Atomic coordinates and related structure factors were deposited in the PDB with accession code 8HFD.

### 4.4. Molecular Docking

Using MOE-Dock [54], 8-HQSA, plumbagin, lupenone, palmitic acid, stig-mast-5-en-3-ol, neophytadiene, and citraconic anhydride were docked to EcALLase-BL21 (PDB ID 8HFD) for their binding capacity. Their S scores and binding modes were compared. The top-ranked confirmations were analyzed.

## 5. Conclusions

Here, we solved the crystal structure of EcALLase-BL21 at a resolution of 2.07 Å (PDB ID 8HFD) to obtain more information for structural analyses. As compared with the previous work, the two missed active site loops in EcALLase-K12 were clearly determined in our structure of EcALLase-BL21. Thus, this current EcALLase structure may be more suitable for use in further docking and product design experiments. In addition, EcALLase-BL21 shared active site similarity with HYDase, an important biocatalyst for industrial production of semisynthetic penicillin and cephalosporins. Based on this structural comparison, the crucial amino acid residues on the dynamic loops were suggested and might be modulated by rational design for the substrate specificity of EcALLase-BL21 for further biotechnological and pharmaceutical applications. The structure of these two dynamic loops solved in this study will significantly affect the docking results, protein engineering strategy, and further understanding of the substrate/inhibitor binding mechanism of ALLase.

## Figures and Tables

**Table 1:** Data collection and refinement statistics.

Data Collection	
Crystal	EcALLase-BL21
Wavelength (*Å*)	1
Resolution (*Å*)	30–2.07
Space group	C_1_2_1_
Cell dimension (*Å*)	*a* = 203.16 *α* = 90°
	*b* = 77.14 *β* = 100.8°
	*c* = 144.85 *γ* = 90°
Completeness (%)	99.02 (97.21) *
<I/σ_I_>	7.39 (2.14)
CC_1/2_	0.992 (0.855)
Redundancy	3.5 (3.2)
Refinement	
Resolution (*Å*)	29.92–2.07
No. reflections	132,804
*R*_work_/*R*_free_	0.208/0.248
No. atoms	
Protein	14,759
Ligands	110
Water	926
R.m.s deviation	
Bond lengths (*Å*)	0.009
Bond angles (°)	1.10
Ramachandran Plot	
In preferred regions	96.89%
In allowed regions	2.66%
Outliers	0.45%
PDB entry	8HFD

* Values in parentheses are for the highest resolution shell. CC_1/2_ is the percentage of correlation between intensities of random half-data sets.

**Table 2:** Results of the docking studies against EcALLase-BL21.

Compound	S Score	Residue	Interaction	Receptor–Ligand Distance (Å)	E (kcal/mol)
8-HQSA	−5.6100	Asn94	H-donor	2.82	−7.4
Plumbagin	−5.2950	Asp315	H-donor	2.94	−3.1
		Asp315	H-donor	3.34	−0.7
Lupenone	−5.7230	No important residue			
Palmitic acid	−5.4744	Ser317	H-donor	3.13	−0.7
		Zn-β	Metal	1.98	−2.8
Stigmast-5-en-3-ol	−6.4260	Asp315	H-donor	3.05	−0.6
		Zn-α	Metal	2.43	−0.9
		Trp332	H-pi	4.50	−1.9
Neophytadiene	−4.6771	No important residue			
Citraconic anhydride	−1.3467	Gln168	pi-H	4.32	−0.6

## Data Availability

Atomic coordinates and related structure factors were deposited in the PDB with accession code 8HFD.

## Supplementary Materials

The following supporting information can be downloaded at: https://www.mdpi.com/article/10.3390/molecules28020827/s1. Figure S1: Structural comparison of EcALLase-BL21 and BsHYDase.
