# Supplementary material for: Crystal Structure of Allantoinase from Escherichia coli BL21: A Molecular Insight into a Role of the Active Site Loops in Catalysis
Source: Molecules. 2023 Jan 13;28(2):827. doi: 10.3390/molecules28020827 (PMC9863593; doi:10.3390/molecules28020827)
Supplement: Supplementary file 1 [file molecules-28-00827-s001.zip › molecules-2137268-supplementary.pdf]

molecules

Supplementary Materials

Crystal Structure of Allantoinase from *Escherichia coli* BL21: A Molecular Insight into a Role of the Active Site Loops in Catalysis

Yen-Hua Huang [1], Po-Chun Yang [1], En-Shyh Lin [2], Ya-Yeh Ho [1], Wei-Feng Peng [1,3,4], Hsin-Pin Lu [1], Chien-Chih Huang [1] and Cheng-Yang Huang [1,5,*]

[1] Department of Biomedical Sciences, Chung Shan Medical University, Taichung City 402, Taiwan
[2] Department of Beauty Science, National Taichung University of Science and Technology, Taichung City 403, Taiwan
[3] Department of Medicine, College of Medicine, Chung Shan Medical University, Taichung City 402, Taiwan
[4] Department of Pediatrics, National Taiwan University Children's Hospital, Taipei 100, Taiwan
[5] Department of Medical Research, Chung Shan Medical University Hospital, Taichung City 402, Taiwan
* Correspondence: cyhuang@csmu.edu.tw

Supplementary Figure

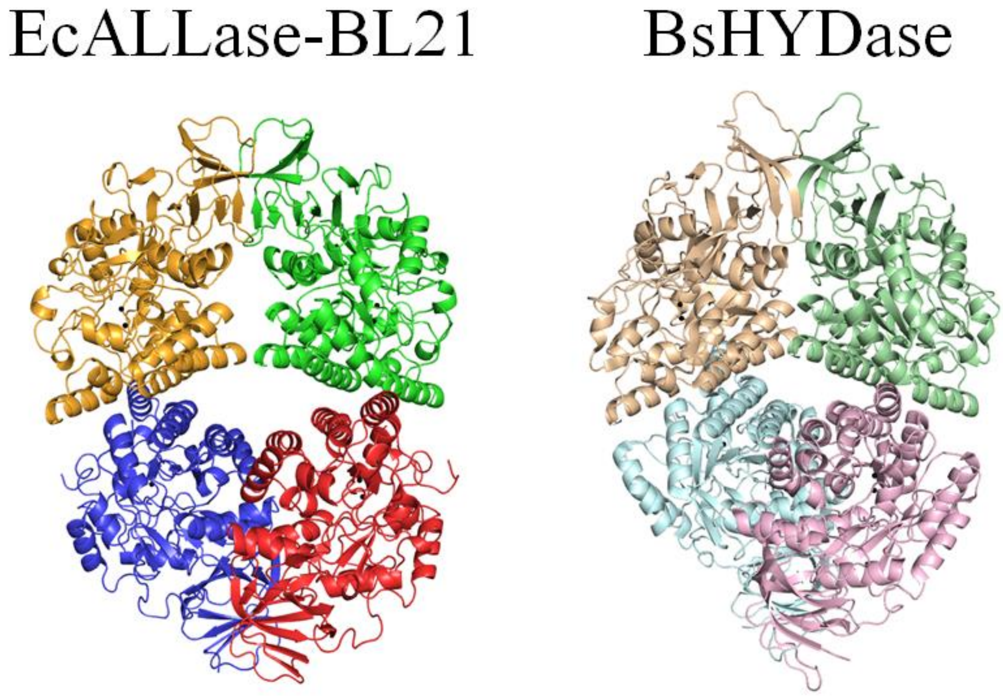

Figure S1. Structural comparison of EcALLase-BL21 and BsHYDase. Ribbon diagrams of EcALLase-BL21 and BsHYDase tetramers. The monomers are colored differently. Two zinc ions in the active site are presented as black spheres. The structure of these two cyclic amidohydrolases has a classic TIM barrel fold and similar active site. However, their substrate specificities do not overlap.
